# Pituitary hormones are specifically expressed in trigeminal sensory neurons and contribute to pain responses in the trigeminal system

**DOI:** 10.1038/s41598-021-97084-y

**Published:** 2021-09-08

**Authors:** Anahit H. Hovhannisyan, Hyeonwi Son, Jennifer Mecklenburg, Priscilla Ann Barba-Escobedo, Meilinn Tram, Ruben Gomez, John Shannonhouse, Yi Zou, Korri Weldon, Shivani Ruparel, Zhao Lai, Alexei V. Tumanov, Yu Shin Kim, Armen N. Akopian

**Affiliations:** 1 Departments of Endodontics, The School of Dentistry, The University of Texas Health Science Center at San Antonio (UTHSCSA), 7703 Floyd Curl Drive, San Antonio, TX 78229-3900 USA; 2 Departments of Oral and Maxillofacial Surgery, The School of Dentistry, The University of Texas Health Science Center at San Antonio (UTHSCSA), San Antonio, TX 78229 USA; 3 Departments of Molecular Medicine, Programs in Integrated Biomedical Sciences and Translational Sciences, The School of Medicine, UTHSCSA, San Antonio, TX 78229 USA; 4 Departments of Microbiology, Immunology and Molecular Genetics, Programs in Integrated Biomedical Sciences and Translational Sciences, The School of Medicine, UTHSCSA, San Antonio, TX 78229 USA; 5 Programs in Integrated Biomedical Sciences and Translational Sciences, The School of Medicine, UTHSCSA, San Antonio, TX 78229 USA; 6 Greehey Children’s Cancer Research Institute, UTHSCSA, San Antonio, TX 78229 USA

**Keywords:** Neuroscience, Physiology, Biomarkers, Diseases

## Abstract

Trigeminal (TG), dorsal root (DRG), and nodose/jugular (NG/JG) ganglia each possess specialized and distinct functions. We used RNA sequencing of two-cycle sorted Pirt-positive neurons to identify genes exclusively expressing in L3–L5 DRG, T10-L1 DRG, NG/JG, and TG mouse ganglion neurons. Transcription factor *Phox2b* and *Efcab6* are specifically expressed in NG/JG while *Hoxa7* is exclusively present in both T10-L1 and L3–L5 DRG neurons. *Cyp2f2*, *Krt18,* and *Ptgds*, along with pituitary hormone prolactin (*Prl*), growth hormone (*Gh*), and proopiomelanocortin (*Pomc*) encoding genes are almost exclusively in TG neurons. Immunohistochemistry confirmed selective expression of these hormones in TG neurons and dural nerves; and showed GH expression in subsets of TRPV1^+^ and CGRP^+^ TG neurons. We next examined GH roles in hypersensitivity in the spinal versus trigeminal systems. Exogenous GH produced mechanical hypersensitivity when injected intrathecally, but not intraplantarly. GH-induced thermal hypersensitivity was not detected in the spinal system. GH dose-dependently generated orofacial and headache-like periorbital mechanical hypersensitivity after administration into masseter muscle and dura, respectively. Periorbital mechanical hypersensitivity was reversed by a GH receptor antagonist, pegvisomant. Overall, pituitary hormone genes are selective for TG versus other ganglia somatotypes; and GH has distinctive functional significance in the trigeminal versus spinal systems.

## Introduction

Sensory ganglia have distinct and specialized physiological and pathophysiological functions^1^. Dorsal root ganglia (DRG) neurons are located in intervertebral foramina at different spinal levels and mainly innervate tissues within the trunk, hands, legs and feet^1^. Trigeminal ganglia (TG) lie within the Meckel's cave and innervate the head and neck area^1^. Sensory neurons of nodose and jugular ganglion complex (NG/JG) are located in the jugular foramen and innervate certain internal organs^1^. Neurons of these ganglia relay sensory information from the external environment as well as internal organs and tissues to the central nervous system^2–4^. Sensory nerves originating from the cell bodies of these ganglia are classified into nociceptive fibers (unmyelinated C fibers and myelinated Aδ) and low-threshold mechanoreceptors (LTMRs; myelinated Aα and Aβ fibers).

Besides their distinct location and innervation pattern, DRG, TG, and NG/JG ganglion sensory neurons each have specialized function with their own respective biochemical and electrical properties. Evidence for these differences has been expanded in recent years with the advent of techniques such as RNA-seq, single-cell sequencing as well as the generation of a set of cell-specific reporter mouse lines^5–7^. These new technologies have confirmed that the proportion of unmyelinated/myelinated trigeminal nerves is substantially lower compared to DRG nerves^8^, while Aα proprioceptors are located in the DRG but absent in TG^9,10^. Single-cell transcriptional profiles of DRG sensory neurons located in T10-L1, which innervate the intestine/colon and feet/legs, are substantially different compared to L3–L5 DRG neurons^11,12^. Moreover, T10-L1 DRG neurons have a unique subset of sensory neuronal groups compared to L3–L5 DRG neurons^11,12^. The DRG neuronal transcriptional profile in turn greatly differs from TG neuronal profiles^13,14^. Additionally, DRG and TG neurons differ in their translational potential for mTOR-related genes and AMP-activated protein kinase^15^. With regards to NG sensory neurons, they possess much different expression profiles compared to sensory neurons from JG and DRG^16^.

RNA-seq of L3–L5 DRG and TG neurons taken from mice expressing the sensory neuron specific advillin-GFP reporter revealed that vasopressin receptor 1A (*Avpr1a*), oxytocin receptor (*Oxtr*), and gamma-aminobutyric acid receptor subunit delta (*Gabrd*) are selectively expressed in TG with little to no expression in DRG sensory neurons^14^. In contrast, DRG sensory neurons have been shown to specifically express a set of *Hox* genes and the receptor for the hormone prolactin (*Prlr*)^14^. Here, we expand upon this experimental approach by including comparison between neurons of DRG from different levels (T10-L1), lumbar DRG, NG/JG and TG. We have used the Pirt/TdTomato mouse reporter line along with enhanced sorting using both a larger nozzle and double sorting methods^13^ that increases purification of sensory neuronal fraction to > 90% while maintaining the natural proportion of large-diameter sensory neurons. Our work, has revealed several additional genes, including prolactin (PRL), growth hormone (GH), and proopiomelanocortin (POMC) with expression restricted to adult male mouse in TG but no other sensory ganglia. Accordingly, we investigated specificity of PRL, GH and POMC expression as well as the functional implication of GH in nociception in the head and neck area.

## Results

**Figure 1:**
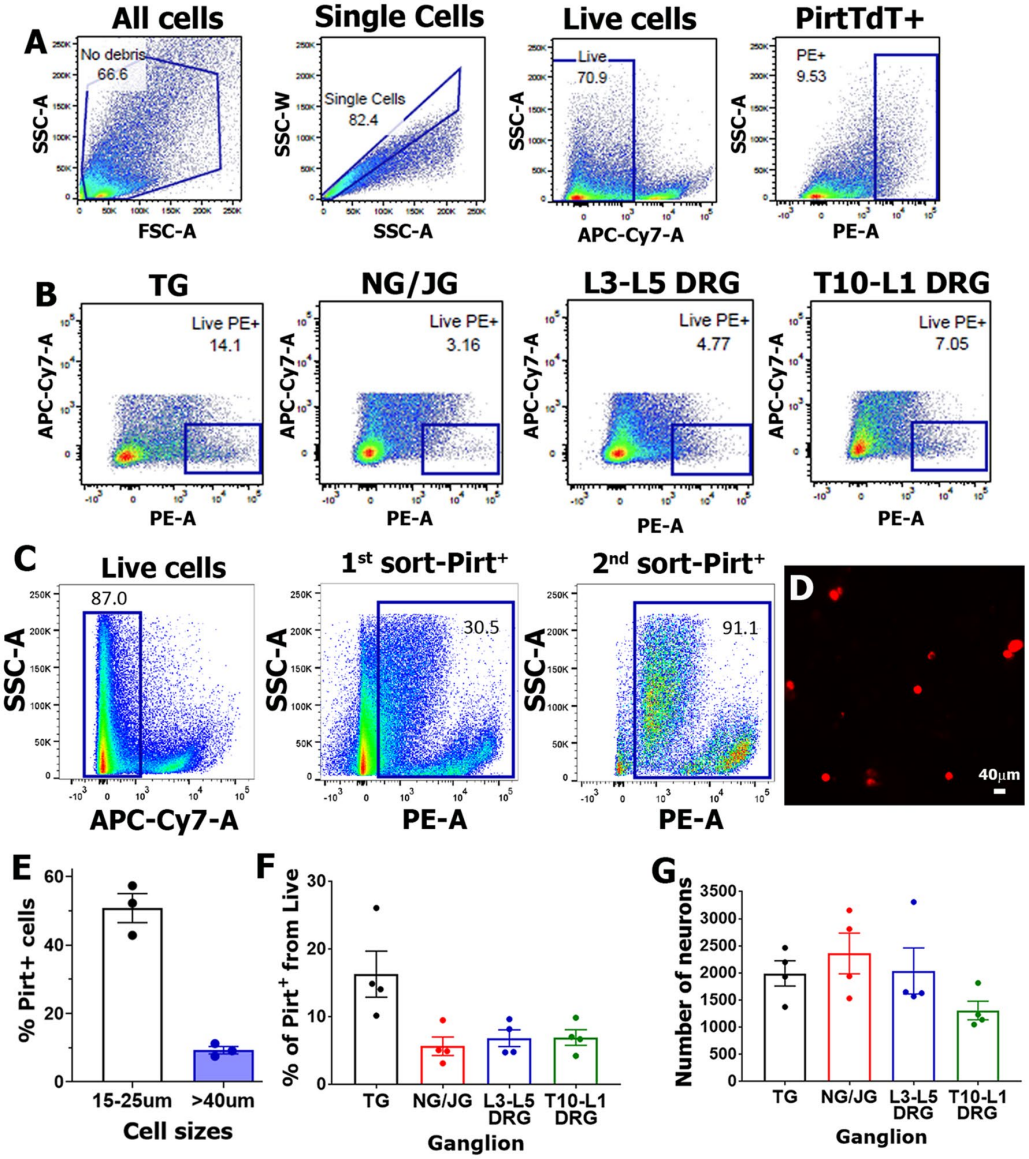
FACS purification of sensory neuronal fractions from ganglia. (**A**) Strategy for sorting of live Pirt/TdTomato^+^ cells from single cell preparation (see details in “Isolation of ganglion sensory neurons” of the “Materials and Methods” section). (**B**) Representative plots of Pirt/TdTomato^+^ live cells at 1st sort for TG, NG/JG, L3–L5 DRG and T10-L1 DRG preparations. (**C**) An example for two cycle sorting and enrichment of a TG sensory neuron fraction. (**D**) Two cycle sorted Pirt/TdTomato^+^ TG neurons plated on a coverslip. A bar corresponds to 40 μm on images captured with 10 × objective. (**E**) Size distributions of Pirt/TdTomato^+^ TG sensory neurons after two cycle sorting. Each sample (n = 1) was generated from independent sorting. (**F**) Percentages of Pirt/TdTomato^+^ cells from all live cells for different ganglia. (**G**) Numbers of sensory neurons in different samples from each ganglion used for RNA isolation and subsequent RNA-seq procedures. Data represented as mean ± SEM. This and all other figures and images were created using Adobe Photoshop Ps (www.adobe.com).

To isolate sensory neurons, we used Pirt/TdTomato reporter mice^17^ and captured small-to-large-sized (10–80 μm) sensory neurons using a 100 μm nozzle. We first gated singlets from doublets (Fig. 1A). Live cells separated from all singlets were used to gate Pirt/TdTomato^+^ cells (Fig. 1A). Medium-to-strongly expressing Pirt/TdTomato^+^ neurons, were gated against TdTomato^-^ wild-type neurons (Fig. 1B). Omitting low expressing Pirt/TdTomato^+^ neurons from gating is a critical step since it increases enrichment levels for Pirt^+^ neurons. Additionally, two cycles of fluorescence-activated cell sorting (FACS) insured maximal purity (> 90%) of sensory neuronal fractions in samples^13^ (Fig. 1C). To validate size distributions of sorted Pirt + neurons, two-cycle FACS-sorted cells were plated on coverslip and their size was assessed using NIS-elements (Nikon Instruments, Melville, NY) (Fig. 1D). TG Pirt^+^ cell size distribution shows that described two-cycle sorting procedure captures small as well as large sensory neurons (Fig. 1E). Proportions of Pirt/TdTomato^+^ cells to live cells were found to vary from ganglion-to-ganglion and between samples (Fig. 1F). TG samples had the greatest cell number with NG/JG samples having the least. However, we used approximately similar numbers of cells for RNA-seq experiments (Fig. 1G).

### Differentially expressing genes (DEG)s for L3–L5 DRG versus T10-L1 DRG sensory neurons

Single-cell sequencing showed substantial differences in the subset of sensory neuronal groups innervating leg and paws (L3–L5 DRG) compared to colon and intestine (T10-L1 DRG) in male mice^11,12^. Accordingly, we compared transcriptomics of sorted sensory neurons from these ganglia. Using strengthened selection criteria outlined in the Materials and Methods section, no DEGs were revealed. However, lowering strength of the selection criteria using fold change (FC) > 5 and *P* value < 0.05 showed that compared to T10-L1 DRG versus L3–L5 DRG had 59 DEGs at RPKM > 5 and 28 DEGs at RPKM > 10. DEG numbers of T10-L1 relative to L3–L5 DRG with the same selection criteria were 58 at RPKM > 5 and 36 at RPKM > 10. Notable DEGs are highlighted in Table 1. Thus, nervous system related genes such as *Ntsr2, Th, Trpv1, Accn1, Kcnh6, Cacna1b,* and *Gabrb3* were enriched in L3–L5 DRG, while immune system related genes *Il6, Ccr1, Cxcl10, Nfkbie, Icam1,* and *Cd248* are mainly expressed in T10-L1 (Table 1). Consequently, gene clustering according to statistical overrepresentation test for biological processes using the PANTHER software assigned 9 predominant DEGs from L3–L5 DRG to the regulation of membrane potential. Biological processes assigned for T10-L1 DRG DEGs were involved in the regulation of MAPK cascade (10 DEGs) and cellular response to organic substance (15 DEGs). Importantly, many of these DEGs, including *Ccr1, Cxcl10, Pdgfc, Nfil3, Irgm1, Il6,* and *Icam1* are linked to immune processes.

**Table 1:** DEGs predominantly present in L3–L5 compare to T10-L1 DRG sensory neurons or vice versa.

Gene Id	T10-L1 DRG RPKM	L3–L5 DRG RPKM	FC	pval	Name
Mpo	0.04025	5.354	126.2	0.04524895	myeloperoxidase
Oprl1	0.2385	9.98875	43.1	0.00053408	opioid receptor-like 1
Ntsr2	0.5095	7.31675	14.7	0.000964351	neurotensin receptor 2
Th	5.67125	68.00275	11.9	0.001879591	tyrosine hydroxylase
Trpv1	13.08425	104.5395	7.9	0.019971988	transient receptor potential cation channel, subfamily V, member 1
Accn1	73.28	574.94525	7.7	0.027897833	acid-sensing ion channel 2
Penk	2.09675	17.1035	7.6	0.000837962	preproenkephalin
Hoxa10	1.1555	8.6405	7.2	0.000480933	homeobox A10
Kcnh6	3.52875	23.24925	6.5	0.048616608	potassium voltage-gated channel, subfamily H, member 6
Cacna1b	1.038	6.65775	6.3	0.02105609	calcium channel, voltage-dependent, N type, alpha 1B subunit
Gabrb3	1.18175	6.41025	5.3	0.008710151	gamma-aminobutyric acid (GABA) A receptor, subunit beta 3
Ikzf4	9.468	0.2785	35.7	0.000209083	IKAROS family zinc finger 4
Il6	12.001	0.961	13.1	0.000966736	interleukin 6
Ccr1	8.6385	0.816	11.8	0.027311494	chemokine (C–C motif) receptor 1
Irgm1	149.1615	17.618	9.0	0.015144517	immunity-related GTPase family M member 1
Cxcl10	272.87475	42.64225	6.7	0.002852945	chemokine (C-X-C motif) ligand 10
Nfil3	125.266	19.62825	6.7	0.00730765	nuclear factor, interleukin 3, regulated
Atf3	1059.589	182.201	6.1	0.000716909	activating transcription factor 3
Nfkbie	32.08375	5.72075	6.0	0.001886081	nuclear factor of kappa light polypeptide gene enhancer in B cells inhibitor, epsilon
Mlf1	5.3825	0.94475	6.0	0.026136752	myeloid leukemia factor 1
Emp1	93.1805	19.0025	5.2	0.000165979	epithelial membrane protein 1
Icam1	41.339	8.8805	5.1	0.019133475	intercellular adhesion molecule 1
Cd248	11.12975	2.389	5.1	0.000807134	CD248 antigen, endosialin

### DEGs for NG/JG versus L3–L5 DRG and T10-L1 DRG

**Figure 2:**
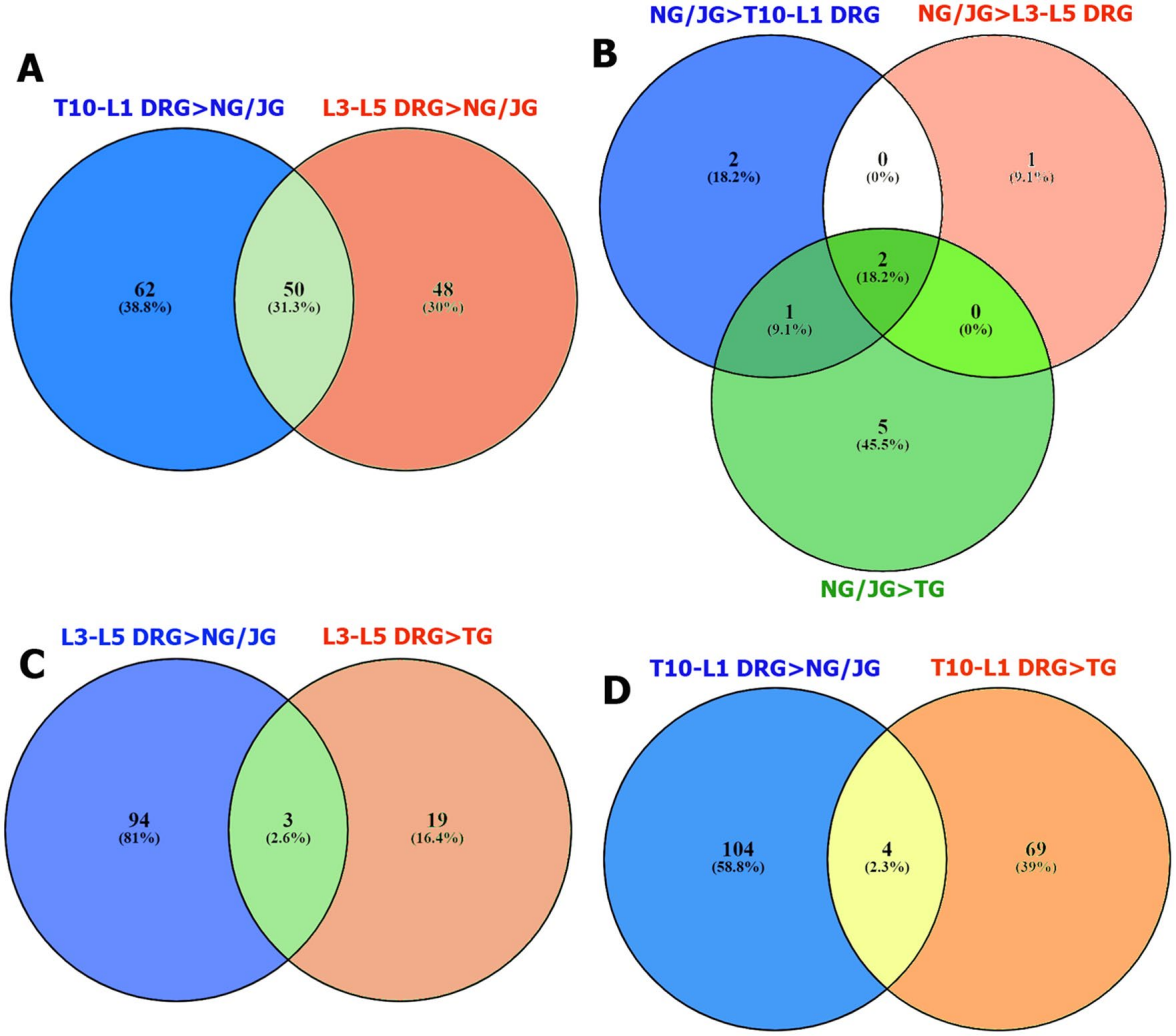
Venn diagrams for T10-L1, L3–L5 DRG and NG/JG sensory neuronal DEGs. Sorted sensory neurons from a variety of ganglia were used for RNA-seq. Venn diagram shows sensory neuronal DEGs predominantly expressed in (**A**) T10-L1 DRG or L3–L5 DRG compared to NG/JG; (**B**) NG/JG compared to T10-L1 DRG, L3–L5 DRG or TG; (**C**) L3–L5 DRG compared to NG/JG or TG; and (**D**) T10-L1 DRG compared to NG/JG or TG.

Nodose-jugular ganglion complex has a specialized role in regulation of several vital visceral organs such as heart, lung, trachea, esophagus, and intestine^16^. Comparison of NG/JG sensory neuronal transcriptomic profiles to DRG revealed only several NG/JG-selective DEGs using outlined selection criteria (see “Materials and Methods”) (Table 2). DRG sensory neurons contain much more predominant DEGs compared to NG/JG. Thus, T10-L1 DRG sensory neurons have 113 DEGs at RPKM > 5 compared to NG/JG; L3–L5 DRG sensory neurons contain 99 such DEGs with 50 overlapping (Fig. 2A). These DRG-selective DEGs relative to NG/JG sensory neurons cannot be broken onto biological processes using statistical overrepresentation test. Nevertheless, DRG has several notable selective DEGs compared to NG/JG complex sensory neurons, including *Mrgprd* and *Hoxa7* (Table 3).

**Table 2:** DEGs predominantly present in NG/JG compare to L3–L5 and T10-L1 DRG sensory neurons.

Gene Id	T10-L1 DRG RPKM	NG/JG	FC	padj	Name
2410057H14Rik	0.1875	94.97825	524.7	0.020384839	NA
Phox2b	0.055	23.26825	465.15	2.51435E−06	paired-like homeobox 2b
Efcab6	0.0745	11.109	169.6	0.00022549	EF-hand calcium binding domain 6
Ddc	0.109	9.39	94.0	0.038644539	dopa decarboxylase
Hoxb5	3.69325	66.943	19.7	0.011614601	homeobox B5

Gene Id	L3–L5 DRG RPKM	NG/JG	FC	padj	Name
Phox2b	0.113	23.26825	234.4	0.000262001	paired-like homeobox 2b
Efcab6	0.278	11.109	46.7	0.006521809	EF-hand calcium binding domain 6
Cgn	0.2505	5.0385	23.5	0.04413522	cingulin

**Table 3:** A partial list of DEGs predominantly presented in L3–L5 and T10-L1 DRG compare to NG/JG sensory neurons.

Gene Id	NG/JG	T10-L1 DRG RPKM	FC	padj	Name
Pdgfrl	0	14.79675	inf	0.00039352	platelet-derived growth factor receptor-like
Cd248	0.011	11.12975	1000.1	0.000144748	CD248 antigen, endosialin
***S100a8***	***0.5705***	***413.61325***	***670.2***	***6.96639E-05***	***S100 calcium binding protein A8 (calgranulin A)***
***S100a9***	***0.3005***	***203.97425***	***636.3***	***2.41717E-06***	***S100 calcium binding protein A9 (calgranulin B)***
Cfb	0.0125	7.03575	406.6	0.005225064	complement factor B
***Mrgprd***	***0.282***	***54.6245***	***183.9***	***5.49484E-05***	***MAS-related GPR, member D***
Pdgfd	0.0385	5.71475	139.4	0.000510529	potassium channel, subfamily V, member 1
***Hoxa7***	***0.3705***	***15.24875***	***38.1***	***0.017499967***	***homeobox A7***

Gene Id	NG/JG	L3–L5 DRG RPKM	FC	padj	Name
Hoxa10	0	8.6405	inf	0.000423477	homeobox A10
Kcnk12	0	7.58575	inf	0.018391471	potassium channel, subfamily K, member 12
Kcnj3	0	6.0835	inf	0.038077184	potassium inwardly-rectifying channel, subfamily J, member 3
Irf5	0.0145	10.098	626.2	0.040860172	interferon regulatory factor 5
Hoxa9	0.01075	7.03575	599.7	0.002559265	homeobox A9
***Mrgprd***	***0.282***	***124.874***	***410.5***	***0.001419568***	***MAS-related GPR, member D***
Kcnv1	0.0135	5.92025	384.3	0.006718591	potassium channel, subfamily V, member 1
Oprl1	0.078	9.98875	116.3	0.016465571	opioid receptor-like 1
***Hoxa7***	***0.3705***	***16.80775***	***41.5***	***0.019179699***	***homeobox A7***
Kctd18	0.2895	10.0905	32.1	0.047190644	potassium channel tetramerisation domain containing 18

DEGs common for T10-L1 DRG > NG/JG and L3–L5 DRG > NG/JG are presented in bold italic.

### DEGs with strong specificity for DRG, NG/JG and TG sensory neurons

Using pair comparison and Venn diagram analysis we found *Phox2b* (paired-like homeobox 2b) and *Efcab6* (EF-hand calcium binding domain 6) as DEGs that were strongly specific to male mouse NG/JG sensory neurons with little-to-no expression in DRG and TG (Fig. 2B; Table 2). *Phox2b* is a transcription factor specifically expressed in neurons of the peripheral and central nervous system^18^. *Phox2b* controls the development of peripheral chemoreceptors and afferent visceral pathways^19^. *Phox2b* is critical for the switch during embryo development from somatic to visceral cranial sensory pathways^20^. The function of *Efcab6* in sensory neurons is not clear. Several mRNA sensory neuronal markers found in the DRG and TG neurons are absent in NG/JG. For example, NG/JG sensory neurons do not express *Mrgprd *(Table 3), an established marker of IB4^+^ DRG neurons^11,21^. A marker of proprioceptors, *Pvalb*, is also absent in NG/JG neurons, but is highly expressed in TG neurons, despite the fact that TG do not have proprioreceptors^1,11,22^. Another example is calcitonin-related polypeptide beta, *Calcb*, which is at > 40-fold lesser in NG/JG sensory neurons compared to DRG or TG. Other notable DEGs lacking expression in NG/JG neurons are *Pdgfrl*, *Orai1* and *Xylt2 *(Table 3).

Hox genes play critical roles in development of many cell types, especially a subset of neurons, during embryogenesis^23^. We found *Hoxa7, Hoxa9,* and *Hoxa10* were selectively expressed in L3–L5 DRG compared to NG/JG or TG (Fig. 2C; Table 3). Comparison of DRG sensory neuron selective expression relatively to only TG sensory neurons found *Hoxb2*, *Hoxb5,* and *Hoxb7* as L3–L5 DRG sensory neuron-specific DEGs. Evaluation of T10-L1 DRG sensory neuronal transcriptomic profiles relatively to NG/JG or TG outlined 4 DEGs, including *Hoxa7* (Fig. 2D). *Hoxb2* and *Hoxb7*, but not *Hoxb5* are also specific for T10-L1 DRG sensory neurons compared to TG neurons. Interestingly most Hox genes, except for *Hoxa7*, did not differentially express in T10-L1 DRG when compared to NG/JG sensory neurons. Overall, our data show that *Hoxa7* was the only DEG distinctively expressed in both T10-L1 and L3–L5 DRG, but not NG/JG or TG. We did not find Hox genes that lack transcription in DRG neurons.

**Figure 3:**
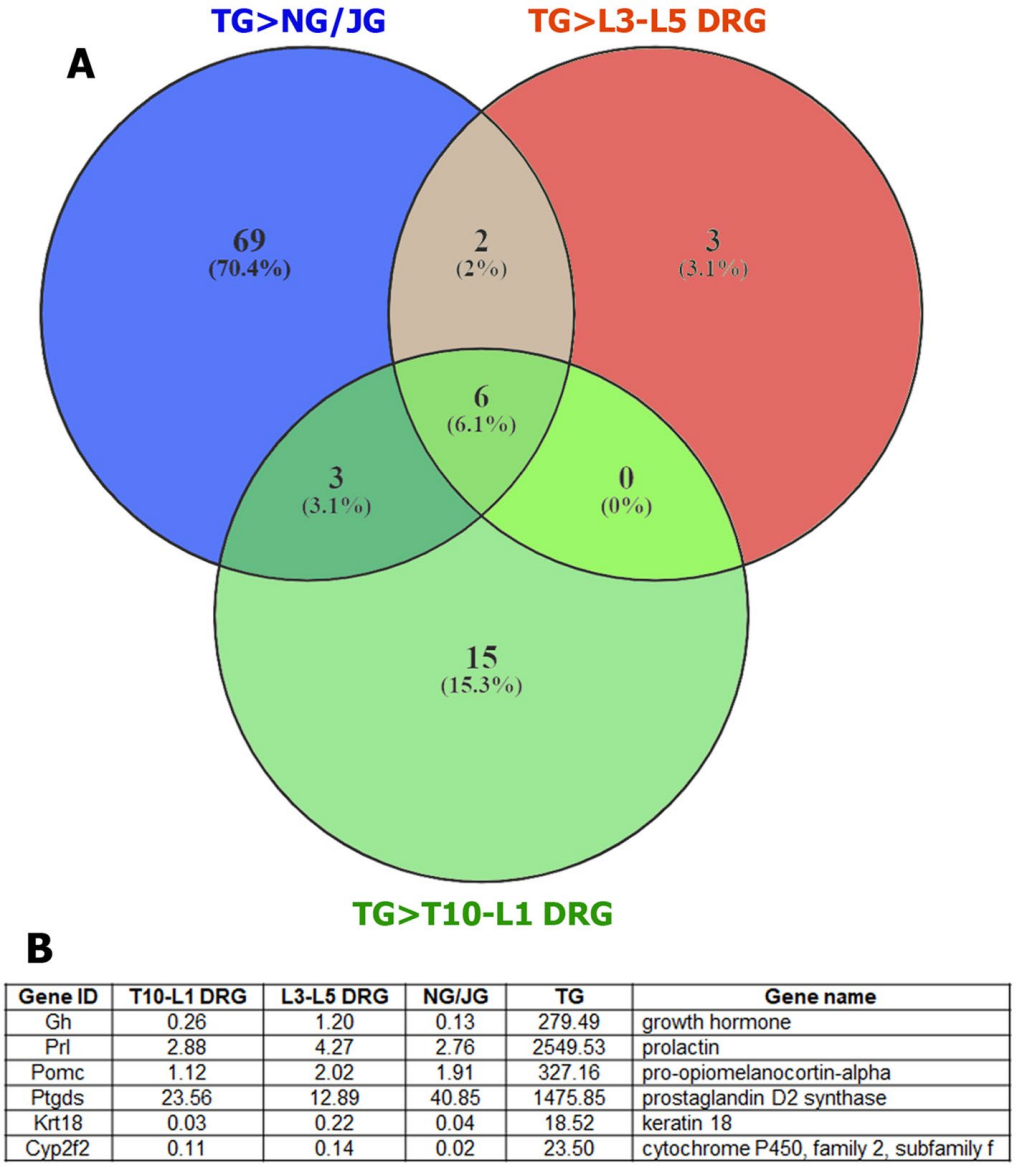
TG sensory neuronal specific genes. (**A**) Venn diagram reveals DEGs specifically expressed in TG compared to L3–L5 DRG, T10-L1 DRG and NG/JG sensory neurons; (**B**) Mean expression levels (in RPKM) for 6 genes in sensory neurons isolated from different ganglia. N = 4 for each ganglion.

Venn analysis of DEGs showed that 6 genes had significantly higher presence in TG compared to L3–L5 DRG, T10-L1 DRG as well as NG/JG sensory neurons (Fig. 3A,B). *Cyp2f2* gene product is critical in the metabolism and toxicity of numerous xenobiotic compounds^24^. *Ker18* plays a role in intestinal pathology^25^ and is linked to peripherin, a well-known marker for small-diameter sensory neurons^26^, located in chromosome 12^27^. *Ptgds* is a key enzyme in prostaglandin synthesis and specifically translated in female lumbar DRG neurons^28^. Accordingly, the PTGDS inhibitor, AT-56 produces hypersensitivity in male but is only effective at high doses in female mice^28^. *Prl, Gh,* and *Pomc* genes encode classical master-hormones, which are highly expressed in the pituitary^29,30^. PRL contribution in sex-dependent pain has been proposed in several studies^31–34^. These studies have mainly focused on expression and function of PRL receptor (Prlr) in DRG or TG neurons^35–38^. Exogenous GH plays an anti-nociceptive role in the spinal system^39^. POMC, which undergoes post-translational processing into multiple peptides including alpha, beta and gamma melanocyte-stimulating hormones (MSH), and adrenocorticotropin (ACTH), is also involved in anti-nociception in the spinal system due to opioids processed from POMC^40^. Further analysis of RNA-seq data showed that several DEGs are in DRG or NG/JG, but not TG sensory neurons. These genes are *Map3k12, Slc35c1, Slc35b4, Ranbp6, Rab9b, Rapgef5, Tspan12, Ggta1,* and *Coro1c*. Involvement of these genes in nociceptive pathway is unknown.

### Expression of Prl, Gh and Pomc gene products in sensory ganglia.

**Figure 4:**
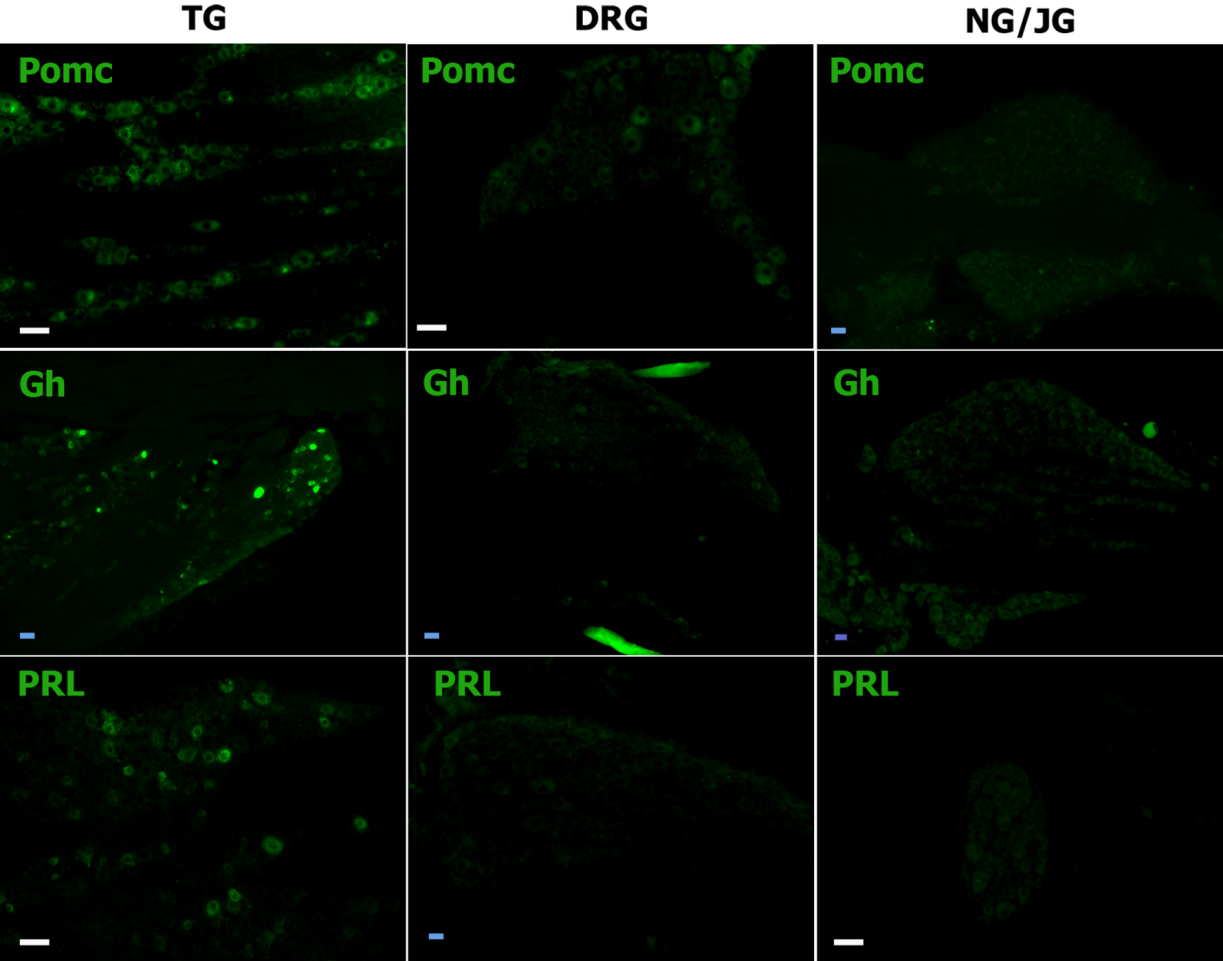
Expression of Prl, Gh and Pomc gene products in DRG, NG/JG and TG. TG, DRG and NG/JG cryo-sections harvested from naïve mice and labeled with Pomc (*upper panels*), Gh (*middle panels*) and Prl (*bottom panels*) antibodies. Type of sensory ganglion is noted above upper panel. White bars correspond to 40 μm on images captured with × 20 objective. Blue bars correspond to 40 μm on images captured with 10 × objective.

**Figure 5:**
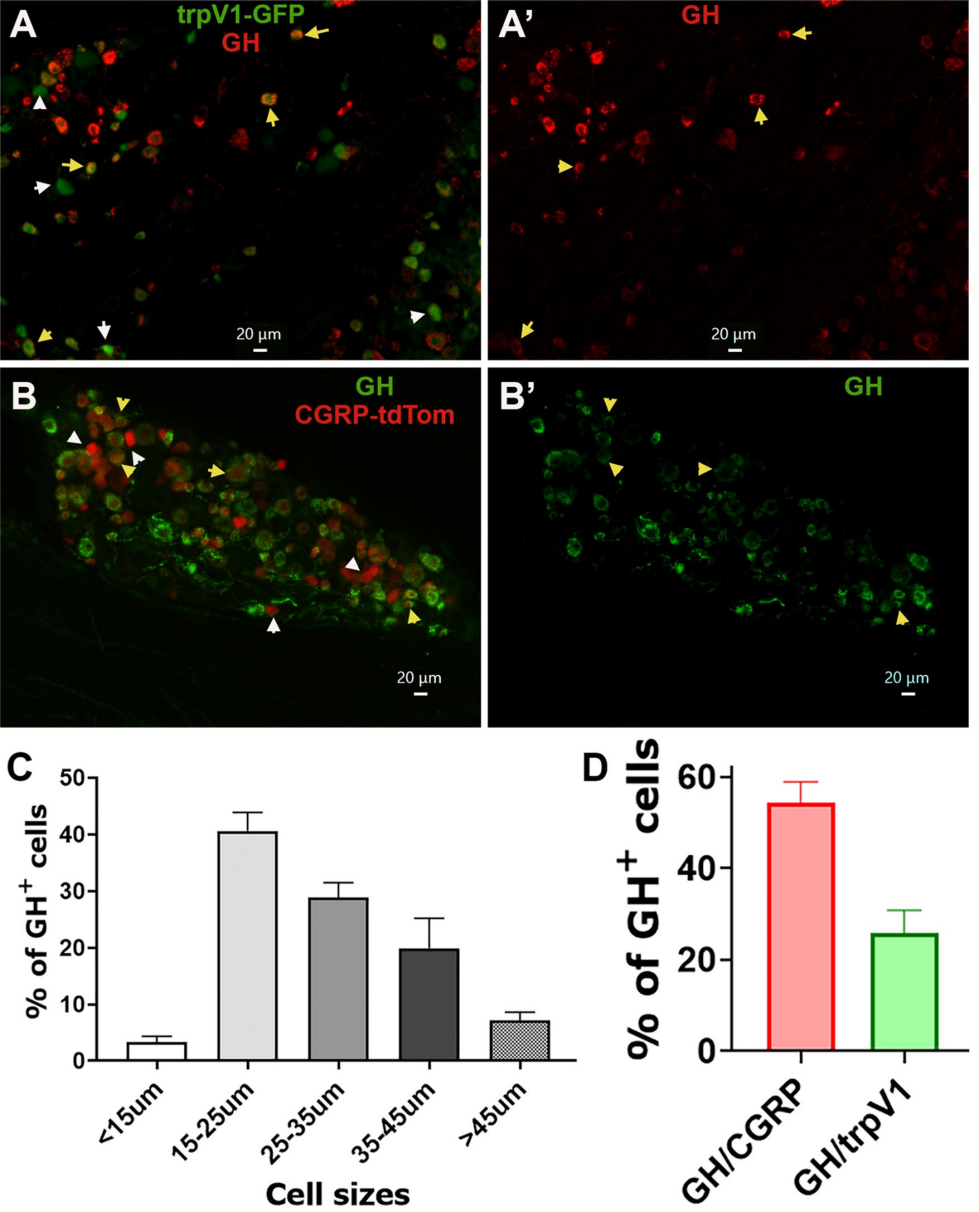
Size distribution of GH^+^ cells in TG and co-expressions of GH with CGRP/TdTomato^+^ and TrpV1-GFP^+^ TG neurons. (**A** and **A’**) Immunohistochemistry (IHC) of TG section from TrpV1-GFP reporter mouse with GH (red). Yellow arrows show TrpV1^+^/GH^+^ cells and white arrows TrpV1^+^/GH^-^ cells. White bars correspond to 20 μm on images captured with 20 × objective. (**B** and **B’**) IHC of TG section from CGRP^cre-ER^/TdTomato reporter mouse with GH (green). Yellow arrows show CGRP^+^/GH^+^ cells and white arrows CGRP^+^/GH^-^ cells. White bars correspond to 20 μm on images captured with 20 × objective. (**C**) Cell size distribution of GH + cells in TG of three independent mice. (**D)** Percentages of GH^+^ cells co-expressing with CGRP^cre-ER^/TdTomato and trpV1-GFP-positice TG sensory neurons.

Sorting pure sensory neuronal fraction is challenging, as sensory neurons constitute only 5–10% of ganglion cells^22,41^, and non-neuronal cells are difficult to dissociate from neurons^41^. Furthermore, RNA-seq data need validation with immunohistochemistry (IHC), as RT-PCR alone again requires FACS sorting of sensory neurons. Accordingly, we used IHC to examine sensory ganglia from male mice expressing PRL, GH and POMC proteins. Here we employed POMC antibodies against 138–150 amino acid residue peptides, which corresponds to the position of a-MSH and ACTH hormone. POMC showed a moderate-to-high level of expression in 63.3 ± 14.2% (n = 3) TG neurons, low level in DRG neurons and was absent in NG/JG neurons (Fig. 4*, the upper panel*). GH (Fig. 4*, the middle panel*) and PRL (Fig. 4*, the bottom panel*) were almost exclusively in a subset of male mouse TG neurons. GH was expressed at medium-to-strong levels (i.e. clear above background) in 17.5 ± 5.4% (n = 3), and PRL in 14.3 ± 6.1% (n = 3) of male mouse TG neurons. These results correspond with previous single-cell sequencing studies of male mouse DRG neurons showing low levels (1 to 9 RPKM) of POMC mRNA, and no expression of GH or PRL mRNA^11^. We also evaluated a GH^+^ cell size distribution and expression in TRPV1^+^ and CGRP^+^ nociceptive sensory neurons. GH was detected in all sizes of sensory neurons with minimal in < 15 μm neurons (3.3 ± 1.1%; n = 3) and maximal presence in 15–25 μm neurons (40.6 ± 3.3%; n = 3; Fig. 5C). To detect TRPV1^+^ and CGRP^+^ TG neurons, TRPV1-GFP and CGRP/TdTomato reporter mice were used^22^ (Fig. 5A,A’,B,B’). Among GH^+^ TG neurons, 26.0 ± 4.9% (n = 3) had TRPV1 (yellow arrows; Fig. 5A,A’,D) and 54.4 ± 4.6% (n = 3) expressed CGRP (yellow arrows; Fig. 5B,B’,D). According to these results, approximately 74% of sensory neurons were GH^+^/trpV1^-^ and 45% GH^+^ TG neurons did not express CGRP. Overall, these data indicate that GH is expressed in both nociceptive and non-nociceptive TG sensory neurons.

**Figure 6:**
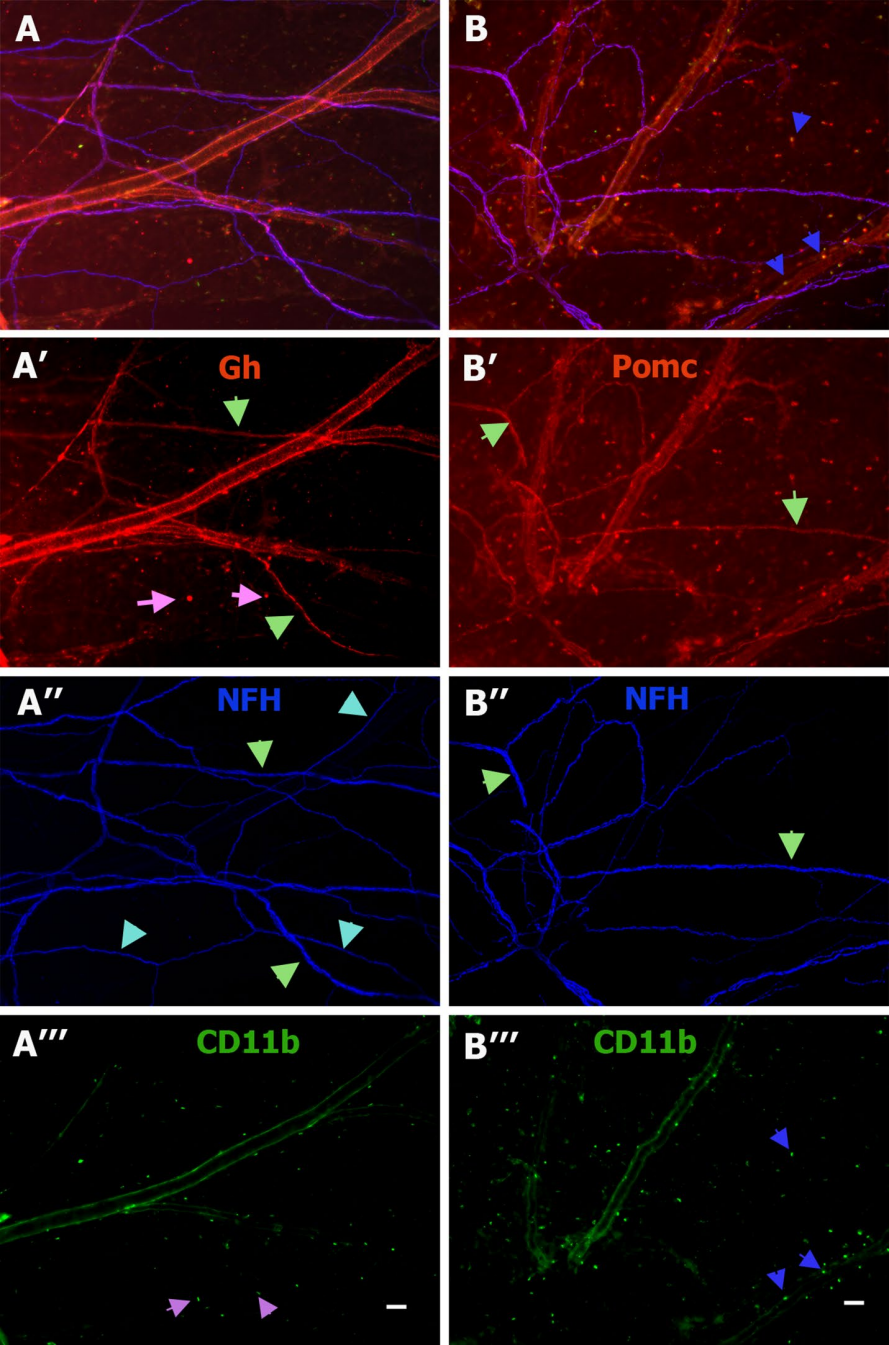
Expression of GH and Pomc in male mouse dura mater. IHC on naïve male mouse dura matter with Gh (red), NFH (blue) and CD11b (green) (**A**), Gh (**A’**), NFH (**A’’**) and CD11b (**A’’’**) antibodies. Green arrows show Gh^+^/NFH^+^ nerve fibers (*panels A’* and *A’’*). Cyan arrows show Gh^-^/NFH^+^ nerve fibers (*panels A’* and *A’’*). Pink arrows show Gh^-^/CD11b^+^ cells (*panel A’’’*). IHC on male mouse dura matter with Pomc (red), NFH (blue) and CD11b (green) (**B**), Pomc (**B’**), NFH (**B’’**) and CD11b (**B’’’**) antibodies. Green arrows show Pomc^+^/NFH^+^ nerve fibers (*panels B’* and *B’’*). Blue arrows show Pomc^+^/CD11b^+^ cells (*panels B and B’’’*). Images were captured with × 10 objective. Bar on panels A’’’ and B’’’ correspond to 30 μm.

To further investigate GH and POMC expression, we performed whole mount IHC on male mouse dura biopsies as it was previously done for PRL^38^. GH is present in a set of neurofilament heavy chain positive (NFH^+^) dural fibers (Fig. 6A’,A’’ *green arrows*), blood vessel cells (Fig. 6A,A’) and a subset of CD11b^+^ myeloid cells (Fig. 6A’,A’’’, *pink arrows*). POMC is expressed in all visible NFH^+^ dural fibers (Fig. 6B’,B’’* green arrows*), blood vessel cells (Fig. 6B,B’) and all CD11b^+^ cells (Fig. 6A,A’’’,* blue arrows*). We note that NFH^+^ fibers (i.e. A-fibers) usually travel with C-fiber inside of perineural sheath in dura^42^. Hence, Fig. 6 cannot definitively tell whether GH and POMC are expressed in C- and/or A-fiber containing TG neurons. Altogether, these data suggest a surprising expression of classical pituitary hormones POMC, GH, and PRL in TG, but not DRG or NG/JG sensory neurons.

### Exogenous GH-induced hypersensitivity in the spinal system

Sex-dependent actions of exogenous and endogenous PRL in the spinal and trigeminal system are reported^31,34,38^. Growth hormone receptor^43^ is expressed on DRG or TG neurons^11^ (see also RNA-seq Supplementary data). IHC data (Figs. 4,5,6) indicate that along with their endocrine effects, endogenous GH could exert autocrine or paracrine actions upon release from non-neuronal extra-pituitary cells at periphery (i.e. hindpaws, dura, masseter muscle, etc.), spinal cord, brain stem or TG neurons. Here, we evaluated whether exogenously delivered GH can produce hypersensitivity in the spinal and trigeminal systems.

**Figure 7:**
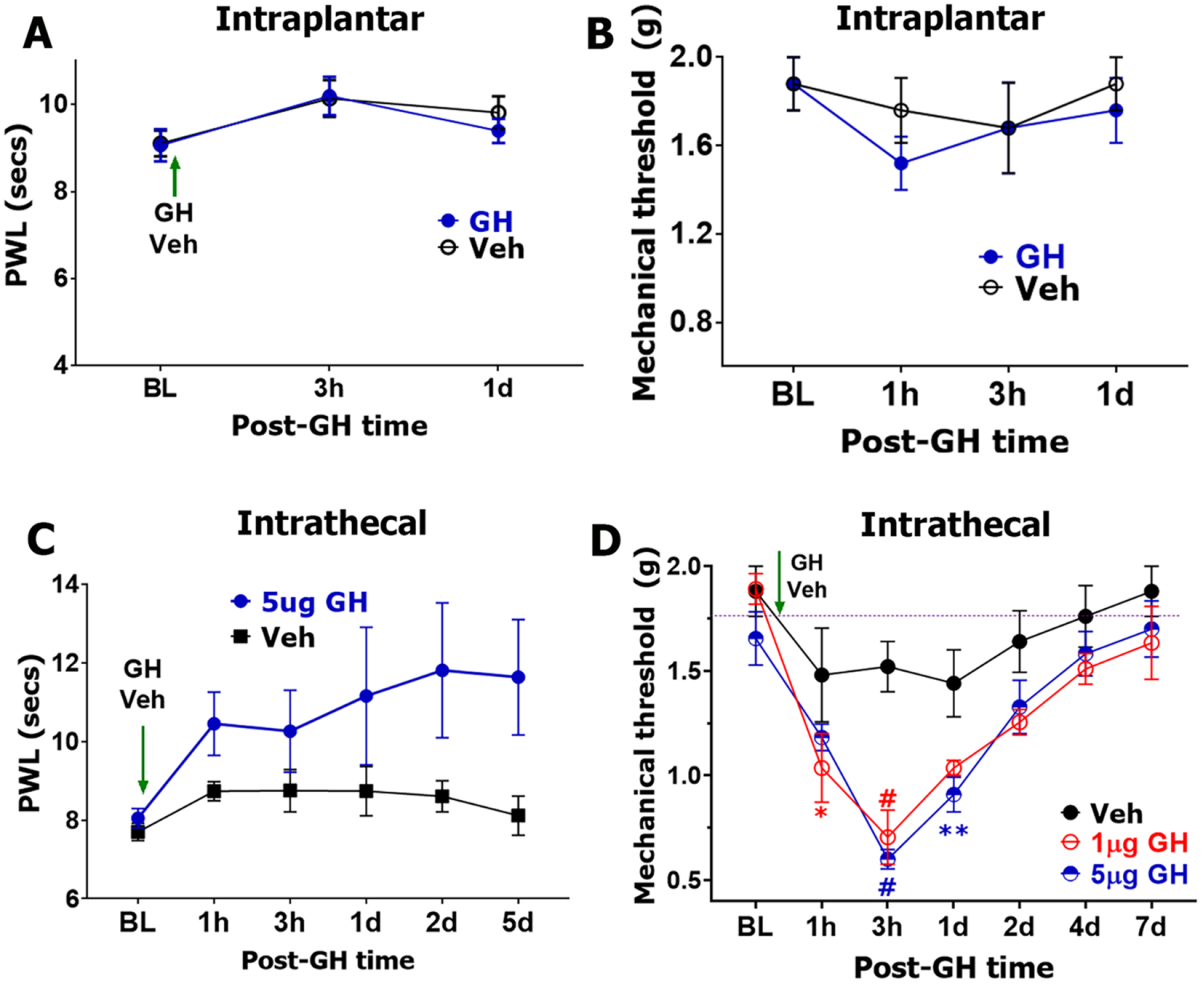
GH-induced acute hypersensitivity in the spinal system. (**A**) Measurement of heat thresholds of hindpaws (as paw withdrawal latency (PWL)) after intraplantar (i.pl.) injection of GH (5 μg) or vehicle (PBS). N = 6. (**B**) Mechanical thresholds of hindpaws after intraplantar (i.pl.) injection of GH (5 μg) or vehicle. N = 5 (**C**) Measurement of heat thresholds of hindpaws (as PWL) after intrathecal (i.t.) injection of GH (5 μg) or vehicle (PBS). N = 5–6. (**D**) Mechanical thresholds of hindpaws after intrathecal (i.t.) injection of indicated dosages of GH or vehicle. N = 6–11. Statistic is 2-way ANOVA, Sidak’s post hoc test (NS—non-significant; **p < 0.01; **p < 0.01; #*p* < 0.0001). Dosages of GH are specified on some panels. Vehicle or GH single administration time point is indicated on every panel with the green vertical arrow. GH delivery route is indicated above each panel.

Intraplantar (i.pl.) injection of 5 μg mouse GH did not generate heat (2-way ANOVA; F (2, 30) = 0.2332; *P* = 0.7934; n = 6; Fig. 7A) or mechanical hypersensitivity (F (3, 32) = 0.2845; *P* = 0.8362; n = 5; Fig. 7B). Intrathecal delivery of 5 μg mouse GH also did not produce thermal hypersensitivity F (5, 49) = 0.7880; *P* = 0.5634; n = 5; Fig. 7C). Moreover, this intrathecal GH effect was slightly anti-hyperalgesic, although statistically insignificant (Fig. 7C). In contrast, two independent trials showed that spinal GH (1 and 5 μg) generated significant mechanical hypersensitivity (F (12, 158) = 1.619; *P* = 0.0911; n = 8–12; Fig. 7D). However, we did not observe dose-dependency of these effects (Fig. 7D). These data suggest that in the spinal system, GH-induced nociceptive responses depend on whether peripheral or central DRG neuron terminals were activated via direct and/or indirect pathways.

### Exogenous GH-induced hypersensitivity in the trigeminal system

**Figure 8:**
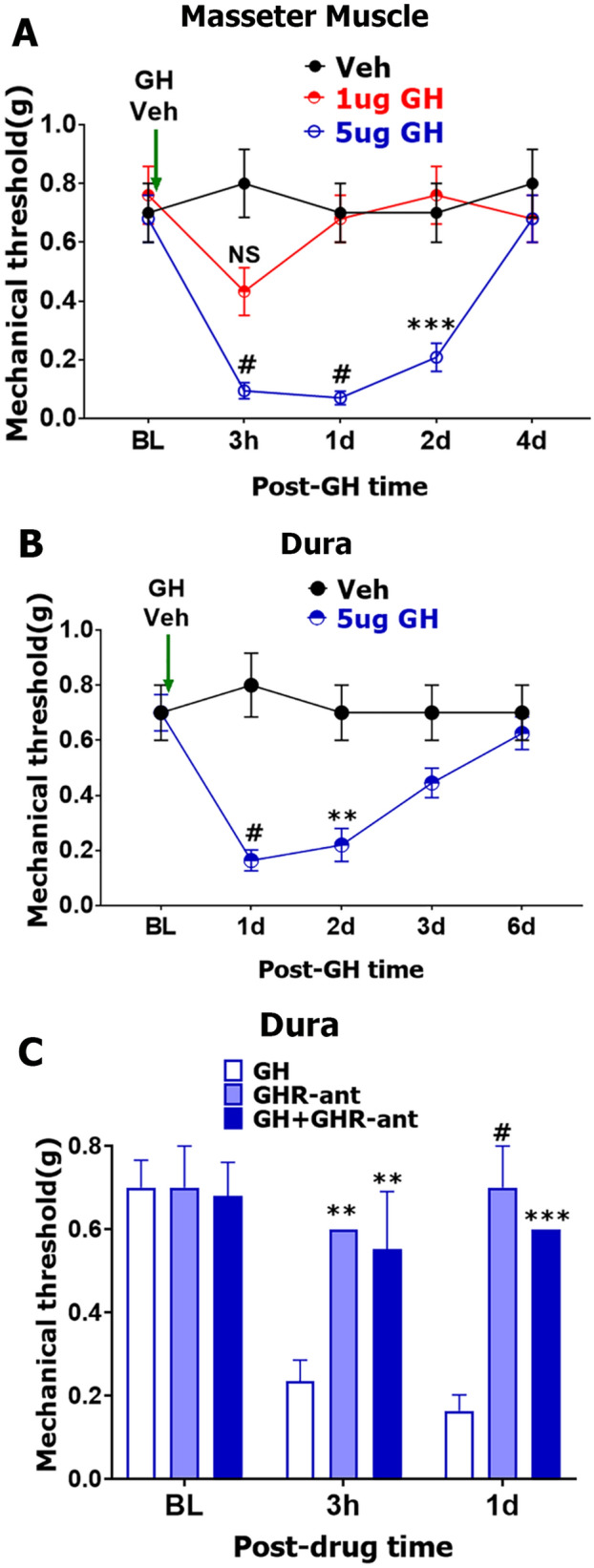
GH-induced acute hypersensitivity in the trigeminal system. (**A**) Orofacial mechanical nociception after intra masseter muscle administration of GH or vehicle. N = 4–5 (**B**) Periorbital mechanical nociception after dura matter administration of GH (5 μg) or vehicle. N = 5–6. (**C**) Periorbital mechanical nociception at 3 h and 1d after dura matter administration of GH (5 μg), GH receptor antagonist (GHR-ant; 25 μm) or a mix of GH (5 μg) and GHR-ant (25 μg). N = 4–8. Statistic is 2-way ANOVA, Sidak’s post hoc test (NS—non-significant; **p < 0.01; ***p < 0.001; #*p* < 0.0001). Dosages of GH are specified on some panels. Vehicle or GH a single administration time point is indicated on A and B panels with the green vertical arrows.

Single administration of exogenous GH into the masseter muscle produced up to 4-days-lasting orofacial mechanical hypersensitivity in a dose-dependent manner (2-way ANOVA; F (4, 35) = 7.284; *P* = 0.0002; n = 4–5; Fig. 8A). Similarly, delivery of GH (5 μg), but not vehicle (PBS) onto dura mater evoked mechanical hypersensitivity in the periorbital area (2-way ANOVA; F (4, 46) = 6.27; *P* = 0.0004; n = 5–6; Fig. 8B). Moreover, these GH-induced pain responses were mediated by GH receptor (GHR), since clinically used GHR antagonist, pegvisomant, which blocks both mouse and human GHR^44,45^, significantly reverse GH effects (F (4, 42) = 4.762; *P* = 0.0029; n = 4–8; Fig. 8C). Overall, GH-induced hypersensitivity in male mice depends on modality (thermal vs. mechanical), sensory system (spinal vs. trigeminal), and application site (peripheral vs. central terminals).

## Discussion

The various sensory ganglia have distinct anatomical locations and unique functions as well as discrete pain pathological conditions associated with their sensory neurons^1–4^. Therefore, characterization of sensory neuron transcription and protein profiles in different ganglia are critically important for understanding underlying mechanisms of pain conditions associated with these sensory ganglia. There are multiple published studies delineating the differences in transcriptional and translational profiles between lumbar DRG and TG in rodents and humans^14,15,41,46–48^. In our and others estimation, sensory ganglia are composed of 90–95% non-neuronal cells (Fig. 1C)^13,14^. Hence, to truly delineate transcriptomic profiles for sensory neurons, there is a need for vigorous and meticulous purification of sensory neuronal fractions from sensory ganglion preparations. Meticulous sensory neuronal purifications by FACS or sensory neuronal ribosome isolation have been performed in some^13–15,41^, but not all studies^46–48^. Sensory neuronal fraction purification by FACS requires the use of reporter mice^14^ or back-labeling of sensory neurons with fluorescent tracers^13^. Reporter mice must possess high specificity in all (or almost all) sensory neurons. Specificity of the advillin promotor (Avil-GFP)^14^ has been disputed^49^. Accordingly, we selected Pirt-cre/TdTomato reporter, specificity of which has been confirmed by two independent differential screening of subtractive libraries^21,50^ and detailed anatomical studies^17,51^. Another challenge in purification of sensory neurons is created by their large size (15–70 μm). To avoid missing large neurons and damaging medium-to-large neurons, 100–130 μm nozzle is required during FACS^13^ (Fig. 1D,E). However, having a large nozzle, in turn, undermines FACS efficiency. Thus, a minimum of two cycles of sorting (used here) is required (Fig. 1C). Moreover, to achieve high enrichment, weakly Pirt-expressing neurons should be omitted by gating (Fig. 1B,C). Next, it appears that ganglion non-neuronal cells have tendency to create doublets with sensory neurons^41^. Separation of doublets from singlets during FACS is paramount for accurate purification of sensory neurons (Fig. 1A). Finally, preparation from wild-type mouse sensory ganglia is necessary for correct setting of gates (Fig. 1B). All these precautions are important for accurate and highly enriched sensory neuron-specific purifications.

We have detected differences in gene expression between sensory neurons of different ganglia by using RNA-sequencing on population of Pirt^+^ neurons. Moreover, DEGs selection for further validation, and consideration for functional studies were quite strong: > 5 RPKM, fold-change (FC) > 5 and statistical significance for DEGs as Padj < 0.05. Populational based RNA-sequencing has strong and weak sites. Thus, this approach has strong signal to noise ratio, as well as reproducibility and reliability of results. Drawback of this approach is that for heterogeneous population as Pirt^+^ neurons from different ganglia, it is impossible to tell whether a variation in transcript reads between distinct ganglia could be due to a differential proportion of gene-expressing neurons, or a differential level of transcript production in the gene-expressing neurons. Such questions could be answered by performing single-cell sequencing and analysis of sensory neurons from different ganglia. However, this approach has its own limitations such as difficulty in performing high quality single-cell sequencing on sensory neurons and especially single cell sequencing analysis for differences between two distinct groups of sensory neurons. In any case, transcriptomic data on differences between populations of sensory neurons generated by these approaches require further lengthy functional studies to prove meaningfulness of findings.

Besides traditional targets—L3–L5 DRG and TG, T10-L1 DRG and NG/JG were used as described experiments, as this allows for a broader picture on diversity for sensory neuronal transcriptomic profiles. Additionally, T10-L1 DRG and NG/JG are functionally distinct from L3–L5 DRG or TG neurons^1–4^. There is a set of DEGs, which were predominantly expressed in L3–L5, T10-L1 DRG, NG/JG or TG sensory neurons (Tables 1,2,3, Figs. 2, 3). These DEGs do not cover any particular biological processes according to the statistical overrepresentation test. Nevertheless, some DEGs are almost exclusively expressed in certain sensory ganglia. For example, we found *Phox2b* and *Efcab6* were specifically transcribed in NG/JG neurons compared to other ganglion sensory neurons (Table 2). Function of *Efcab6* is not clear, while *Phox2b* regulates development of viscera controlling neurons of the peripheral and central nervous systems^18–20^. Moreover, NG/JG did not have sensory neurons expressing well-known markers—*Pvalb* and *Mrgprd* (Table 3). Hox genes are selective for DRG sensory neurons. *Hoxa7* is specific for L3–L5 as well as T10-L1 DRG compare to NG/JG and TG neurons. Additionally, *Hoxa9* and *Hoxa10* express in L3–L5 DRG, but not NG/JG or TG neurons. Finally, *Hoxb2*, *Hoxb5* and *Hoxb7* were found to be specific for L3–L5 DRG neurons when compared to TG neurons. This is in line with others who have reported *Hox* genes exclusive for L3–L5 DRG neurons compared to TG neurons^14^. *Hox6a, Hoxb3, Hoxb6, Hoxc6, Hoxc8*, *Hoxc9, Hoxc10, Hoxd8, Hoxd9,* and *Hoxd10* were all exclusively expressed in L3–L5 DRG neurons, but P adjustment (Padj) was > 0.05 due to low RPKM values for these DEGs. Others have reported L3–L5 DRG sensory neuron-exclusive genes, such as *Kcnq5, Prlr,* and *AW551984*^14^, at higher levels compared to TG neurons. Interestingly, *Prlr* expression is lacking in NG/JG sensory neurons. Our data showed that reported TG sensory neuron-selective genes, such as *Rgs6, Gabrd,* and *Oxtr*^14^, have substantially (3–tenfold) higher RPKM in L3–L5 DRG, T10-L1 DRG, and even NG/JG neurons, while *Avpr1a* is evenly expressed in DRG and TG, but absent in NG/JG neurons. TG sensory-neuron-specific DEGs are presented in Fig. 3B. Functional significance of *Krt18* and *Cyp2f2* gene products for TG neurons are not clear. *Ptgds* and *Prl* genes are involved in female-selective mechanisms of nociception for the spinal system^28,32–34,38^. Involvement of PTGDS or PRL proteins in sex-dependent regulation of nociception in the orofacial region have recently been reported^38^.

Here, we have focused our efforts to investigate whether GH contributes to regulation of nociception in the spinal versus trigeminal system. GH did not exert acute heat and mechanical hypersensitivity in male mouse hindpaw after local administration (Figs. 7A,B). However, intra-spinal (i.e. intrathecal) injection of exogenous GH (1 or 5 μg) produced up to 4-day lasting mechanical, but not heat hypersensitivity (Figs. 7C,D). Interestingly, no dose-dependency of GH action was recorded. This could indicate that lower GH dosage is able to produce hypersensitivity via acting on central terminals of sensory neurons. Another possibility is that GH activates spinal cord cells, which in turn release factors activating or sensitizing the central terminals in the spinal system. Unlike the spinal system, stimulation of peripheral terminal in the trigeminal system by GH produced profound acute hypersensitivity. Thus, orofacial mechanical hypersensitivity was induced by after single administration of GH into masseter muscle and periorbital mechanical hypersensitivity was detected after injection of GH into dura mater of male mice (Figs. 8A,B). The effect of GH in the trigeminal system was dose-dependent (Fig. 8A). This GH-induced hypersensitivity was meditated by GHR and reversed by pegvisomant (Fig. 8C). These data indicate that ability of GH in induction of hypersensitivity in male mice depends on several factors such as modality (thermal vs. mechanical), sensory system (spinal vs. trigeminal), and application site (peripheral vs. central terminals).

Previous study shows that systemic application of GH (0.5 mg/kg) increased baseline mechanical nociception in P7, but not P14 male mice^39^. In contrast, heat baseline nociception was reduced in P7, but not P14 male mice^39^. These results agree with our data (Fig. 7A,B). Interestingly, multiple systemic GH treatments attenuated carrageenan acute inflammatory hypersensitivity^39^. GH release hormone receptor (GHRHR) ablation induced behavioral and afferent hypersensitivity during early developmental stages but resolved at P21 age male mice^52,53^. Reported effects of GH in young male mice are attributed to insulin-like growth factor 1 receptor^39^. It is not clear whether the action of GH is local, in the spinal cord or brain. However, systemically delivered GH is not readily cross the blood brain barrier^54^. Moreover, effect of GH in the spinal system has yet to be assessed in females, especially female mice at the reproduction age. Our data and previous literature suggest that GH could have differential effects on modulation of nociception and hypersensitivity in the trigeminal versus spinal systems. This is also supported by clinical data. Thus, acromegaly patients having excess GH often reported severe and prolonged headaches, but no pain in limbs (see review^55^).

An increased ACTH brings to Cushing and Addison disease. Patients with Cushing’s syndrome and Addison disease seldom report abdominal pain^56,57^. However, pain during these diseases was not associated with elevation of ACTH. Despite PRL, GH, and POMC-derived peptides/proteins are predominantly expressed in the pituitary at high levels, there is evidence for extra-pituitary presence of these hormones, especially in immune cells^58–61^. POMC-derived peptide could be involved in cell–cell communication via autocrine and paracrine mechanisms. Moreover, reduction of already low levels of POMC expression in DRG neurons of female and male mice with diabetic neuropathy contributes to hypersensitivity^40^. This effect was attributed to endorphins that could be processed from POMC^40^. Overexpression of POMC-derived endo-opioids in L3–L4 DRG does not change baseline nociception in female mice but suppresses diabetic neuropathy-induced hypersensitivity^40^. Interestingly, diabetic female mice develop heat hypersensitivity, as opposed to hyposensitivity in males, while POMC-MOR expression is downregulated in both female and male mice, and POMC effects in diabetic mice was not sex-dependent^40^. Like GH, POMC-derived peptides could have distinct signaling pathways and outcomes on modulation of nociception and hypersensitivity in the trigeminal versus spinal systems.

Overall, our approaches identify several DEGs that are specifically expressed in sensory neurons of DRG, NG/JG or TG. Interestingly, these genes include very prominent players in the endocrine system—PRL, GH, POMC as well as prolactin and oxytocin receptors. Moreover, *Prl, Prlr, Pomc* and *Ptgds*, which are involved in differential occurrences of pain disorders in men and women^28,31,34,40,62^, have differential expression for TG versus DRG and NG/JG neurons. Based on our findings, we favor the hypothesis that certain critical proteins for the endocrine system have different signaling pathways as well as pathophysiological outcomes for the spinal versus trigeminal system. Our results advance our understanding of unique properties of sensory ganglion neurons and provide a building step for further studies on regulation of nociceptive pathways by endogenous GH and POMC for pathological pain conditions affecting head and neck area (i.e. the trigeminal system).

## Materials and methods

### Mouse lines and reagents

All animal experiments conformed to APS’s Guiding Principles in the Care and Use of Vertebrate Animals in Research and Training, and to protocols approved by the University Texas Health Science Center at San Antonio (UTHSCSA) Animal Care and Use Committee (IACUC). We followed guidelines issued by the National Institutes of Health (NIH) and the Society for Neuroscience (SfN) to minimize the number of animals used and their suffering. The reporting in the manuscript follows the recommendations in the ARRIVE guidelines.

Eight-to-twelve-week-old naïve C57BL/6J (The Jackson Laboratory, Bar Harbor, ME) male mice were used for all described experiments. In fluorescent activated cell sorting (FACS) experiments, we used Pirt^cre/-^/Rosa26^LSL-tdTomato/+^ (Pirt/TdTomato) reporter mice, which show specific expression of red fluorescent protein, TdTomato in all sensory neurons^63^. In some immunohistochemistry (IHC) experiments, we used CGRP^cre/-ER^/Rosa26^LSL-tdTomato/+^ (CGRP/TdTomato; kindly provided by Dr. Pao-Tien Chuang, UC San Francisco, San Francisco, CA) reporter mice, which show specific expression of TdTomato (red) in CGRP^+^ sensory neurons^22^; and TRPV1-GFP reporter mice (purchased from the GENSAT program; MMRRC services UC Davis, CA), which show specific expression of GFP (green) in TRPV1^+^ sensory neurons^22^.

Mouse GH was kindly provided by Novo Nordisk (Dr. Peter Thygesen). GH receptor (GHR) antagonist (Pegvisomant; PF-04748184), which blocks both mouse and human GHR, was kindly provided by Pfizer (Pfizer’s Compound Transfer Program).

### Isolation of ganglion sensory neurons

Left and right whole L3–L5 DRG, T10-L1 DRG, NG/JG complex and TG tissue biopsies were dissected after perfusion of Pirt/TdTomato mice with phosphate buffer pH 7.3 (PB). Ganglion tissues were used for single-cell suspension generation as previously described^13^. Briefly, whole ganglia were treated with 125 μg/ml liberase (Millipore-Sigma, St. Louis, MO) and 200 μg/ml dispase II (Millipore-Sigma, St. Louis, MO) in Hank’s solution for 60 min. Reaction was stopped by washing tissues with DMEM/L-glutamate/5% fetal bovine serum (FBS) media. Ganglia were dispersed to single-cell conditions by pipette and filtered through 100 μm strainer.

FACS was used to isolate all sensory neurons, which express TdTomato^13^. Consecutive gates were used to isolate Pirt/TdTomato^+^ cells. First, debris was excluded by forward scatter area (FSC-A) and side scatter area (SSC-A) gating. Second, duplets and clumps were excluded by side scatter width (SSC-W) and side scatter area gate (SSC-A) gate. Third, dead cells were excluded by allophycocyanin-Cy7 (APC-Cy7) Zombie NIR Fixable Viability Kit (Biolegend). Forth, Prrt-tdT+ bright cells were gated in PE phycoerythrin (PE-A) channel and sorted. Two cycles of sorting, which provide > 90% purification of sensory neuronal fractions, were conducted on 5 laser FACS Aria-IIIu cell sorter equipped with 100 μm nozzle.

### RNA isolation, cDNA synthesis and RNA-sequencing

RNA was isolated from single-cell sensory neuron suspension using Qiagen RNeasy (Universal Mini Kit) as was previously described^74^. RNA (< 10 ng) quality was accessed after cDNA preparation using Fragment Analyzer Agilent 2100 Bioanalyer RNA 6000 Nano chip (Agilent Technologies, Santa Clara, CA). RNA-seq cDNA libraries from sensory neuronal fraction (3000–35,000 neurons depending on types of ganglia) were prepared using oligo dT according to SMART-seq-2 protocol^75,76^ with previously described modifications^13^. cDNA libraries were subjected to quantification and subsequent 50 bp single read sequencing run with Illumina HiSeq 3000 platform (Illumina, San Diego, CA). Each group have n = 4 samples. Depth of reads was 30–50 × 10^6^ bp for each sample.

### Transcriptomic data analyses and statistics

Sequencing data from all samples were processed in the same way as previously described^13^. Briefly, RNA-seq readings were de-multiplexed with CASAVA and the FastQ files were generated. Raw reads were aligned to mouse genome build mm9/UCSC hg19 using TopHat2 default settings^77,78^. The BAM files obtained after alignment were processed using HTSeq-count to obtain the counts per gene, and then converted to RPKM (Read Per Kilobase of gene length per Million reads of the library)^79^. Differentially expressing genes (DEGs) were identify using DESeq software after performing median normalization^80^. Quality control statistical analysis of outliers, intergroup variability, distribution levels, PCA and hierarchical clustering analysis was performed to statistically validate the experimental data. Multiple test controlling was performed with Benjamini–Hochberg procedure and adjusted *p *value (Padj) was generated. Criteria for selection of DEGs for the further analysis are following: > 5 RPKM, fold-change (FC) > 5 and statistical significance for DEGs as Padj < 0.05. This allows to select DEGs with high levels expression and significant difference in expression levels. DEGs were clustered according to biological processes using the PANTHER software (http://www.pantherdb.org/).

### Immunohistochemistry

Immunohistochemistry (IHC) was performed on L3–L5 DRG, NG/JG complex and TG sections, and dura mater biopsies dissected from naïve male 4% paraformaldehyde-perfused mice. Cryo-section (25 μm) generation and IHC process were performed as described^22,36^. Whole-mount IHC on dura biopsies was carried out. Intact dura was fixed again with 4% paraformaldehyde and cryoprotected with 30% sucrose in phosphate buffer. Labeling with primary and secondary antibodies were done on submerged dura samples in wells of 12-well plates. IHC was simultaneously performed on 4–8 sections generated from 3 animals. The following primary antibodies were used: anti-CGRP rabbit polyclonal (Sigma; C8198; 1:300)^64–66^, anti-neurofilament H (NFH) chicken polyclonal antibodies (BioLegend, San Diego, CA; cat: PCK-592P; 1:400)^67^, anti-PRL rabbit polyclonal antibodies (Bioss, Boston, MA; cat: BS-23763R; 1:200), anti-POMC rabbit polyclonal antibodies (Bioss, Boston, MA; cat: BS-1195R; 1:200), anti-POMC rabbit polyclonal antibodies (Bioss, Boston, MA; cat: BS-1195R; 1:200)^68^ and anti-GH rabbit polyclonal antibodies (FabGennix; Frisco, TX; cat: GH-112AP; 1:200). After labeling with primary antibodies, sections and dura biopsies were incubated with species appropriate secondary antibodies (1:200; Jackson Immuno-Research Laboratories, Inc., West Grove, PA). Control IHC was performed on tissue sections processed as described but either lacking primary antibodies or lacking primary and secondary antibodies.

Images were acquired using a Keyence BZ-X810 All-in-One Fluorescent Microscope (Keyence, Itasca, IL), a Nikon Eclipse 90i microscope (Nikon Instruments, Melville, NY) equipped with a C1si laser scanning confocal imaging system or Zeiss (Carl Zeiss, Jena) LSM single photon confocal microscope. Images were processed with NIS-elements (Nikon Instruments, Melville, NY), ZEN (Carl Zeiss, Jena) or Adobe Photoshop CS2 software. Gain setting was constant during acquisition, and it was established on no primary control slides. Cell counts from IHC images acquired as Z-stack were performed using Image J software. Total cells/section and positive cells were counted. Cell counting were performed independently by two investigators. We used 3 independent mice to generate sections and counted 3–5 sections per mouse. Thus, each group has n = 3, and data for each sample are represented by mean values from 3 to 5 sections generated per animal.

### Dural, masseter muscle, hindpaw (intraplantar) and spinal cord (intrathecal) injections

Injection into hindpaw (i.e. intraplantar) was done as previously described^69^. Briefly, mice were anesthetized with 5% isoflurane (v/v) for ≈20–30 s. The plantar surface of the footpad was cleaned with betadine and 70% ethanol. Solutions with hormones (10 μl) were injected using 1 ml-insuline syringe with 30-gauge needles into the metatarsal region of the hindpaw. Pressure on hindpaw was maintained several seconds after withdrawal of the needle.

Injection into spinal cord (i.e. intrathecal) was done as described^34^. Briefly, tissue above spinal L3–L5 levels was cleaned with betadine and 70% ethanol. The mice were anesthetized with 5% isoflurane (v/v) for ≈1–1.5 min. Injection were performed with 30-gauge 1/2-inch needle mated to a 10-pl luer tip syringe (Hamilton, Reno, NV). The needle is inserted into the tissue above L4 or L5 spinal levels so that it slips into the groove between the spinous and transverse processes. The needle is then moved carefully forward to the intervertebral space as the angle of the syringe is decreased to about 10°. The tip of the needle is inserted so that approximately 0.5 cm is within the vertebral column. Solutions with hormones (10 μl) were injected and the needle rotated on withdrawal.

Injection into masseter muscle were also performed on mice anesthetized with 5% isoflurane (v/v) for 1–1.5 min. The skin over masseter muscle was cleaned with betadine and 70% ethanol. Solutions with hormones (10 μl) were injected using 1 ml-insuline syringe with 30-gauge needles into region closer to tendinous aponeurosis of the superficial head of the masseter muscle.

Administrations of solutions to dura matter were performed according to previously published methods^70^. Briefly, mice were anesthetized under isoflurane for < 2 min and injected with 5 μl hormone solution using a modified internal cannula (Invivo1, part #8IC313ISPCXC, Internal Cannula, standard, 28 gauge, fit to 0.5 mm). The inner portion of the cannula was adjusted with calipers to extend from 0.5 to 0.65 mm in length. The cannulas were inserted through the soft tissue at the intersection of the lambdoidal and sagittal sutures.

### Hypersensitivity testing of the hindpaws, periorbital skin and V2 facial skin area over masseter muscle

All experimenters performing testing on mice and data analysis were done blinded for all behavior experiments. Allocation of animals to treatment groups was randomized by a “blinder” that drew animal numbers from a bag of paper slips. Mice were habituated to the testing environment for at least 1 h prior to nociception measurements on the hindpaw. Heat-induced nociception was measured using an automated Hargreaves’ apparatus as previously described^71^. Mechanical stimulus-induced nociception following the intraplantar injection was assessed by determining paw withdrawal threshold using up-down von Frey method^72^.

Mechanical hypersensitivity in head and neck area was performed on unrestrained animals^70,73^. To reduce any effects of restraint by hands or grasping the tail, mice were habituated^73^. For measurements of mechanical hypersensitivity following masseter muscle injection, sequence of following procedures was carried out. First, naïve mice were placed in a black wire mesh box (4 × 4 × 4″) and allowed to freely move for ≈1 h. This procedure was repeated 3 days. Next, test von Frey filament probing of V2 facial skin area over masseter muscle was applied to each mouse for 3–4 consecutive days. Each grade of von Frey filament was applied 3 times at intervals of a few seconds. The stimulation always began with the filament producing the lowest force and stopped when mice are responded to 3 consecutive stimulations with a graded von Frey filament. A brisk or active withdrawal of the head from the probing filament was defined as a response. Mice with mechanical threshold > 0.6 g at V2 facial skin area, which is considered baseline mechanical nociception, were selected for drug/hormone injection. After injection into masseter muscle, mechanical hypersensitivity was regularly assessed for 1 week.

Headache-like behavior following dura injection were assessed as described^70^. To habituate mice, they were placed in 4 oz paper cups (Choice) for 2 h a day for 3 consecutive days. von Frey testing of the periorbital skin (the midline of the forehead at the level of the eyes), which is used to assess headache-like behavior, was carried out on each mouse located in a paper cup for the 3–4 consecutive post-habituation days. Baselined animals were defined as animals that exhibited a withdrawal threshold > 0.6 g. Mice with a baseline threshold < 0.6 g at the end of 3 habituation days and 4 test days were excluded from experiments. After application of drug/hormone to dura, mechanical thresholds were regularly determined for 1 week by applying von Frey filaments to the periorbital skin in an ascending/descending manner starting from the 0.02 g filament. If the animal responded to this filament, decreasing forces were applied until the 0.008 g filament was reached.

### Statistical analysis

GraphPad Prism 9.0 (GraphPad, La Jolla, CA) was used for statistical analyses. Data in the figures are mean ± standard error of the mean (SEM), with “n” referring to the number of analyzed mice for IHC or behavioral experiments. Statistical changes between 2 or more groups with two variables were analyzed by regular 2-way ANOVA with Sidak’s post-hoc tests. A difference is accepted as statistically significant when *p* < 0.05. Number of animals in a group, interaction F ratios, and the associated *p* values are reported.

### Ethical approval and informed consent

All experimental protocols were approved by the UTHSCSA IACUC committee. Protocol numbers are 20190114AR and 20190083AR.

## Supplementary Information

Supplementary Information 1.

Supplementary Information 2.

Supplementary Information 3.

Supplementary Information 4.

Supplementary Information 5.

Supplementary Information 6.

## Data Availability

RNA-seq data has been deposited to GEO. Accession is GSE168601. Supplementary excel files present the raw gene counts per gene in all our sequencing experiments for Pirt^+^ sensory neurons. These supplementary files are *DRG L3–L5 vs. DRG L1-T10*; *NG/JG vs. DRG L1-T10*; *NG/GJ vs. DRG L3–L5*; *TG vs. DRG L1-T10*; *TG* vs. *DRG L3–L5* and *TG vs. NG/JG*.
